# A Rare Coexistence of Paraneoplastic Cerebellar Degeneration: Papillary Thyroid Carcinoma

**DOI:** 10.3390/curroncol28010055

**Published:** 2021-01-19

**Authors:** Zeynep Özözen Ayas, Gülgün Uncu

**Affiliations:** Eskisehir City Hospital, Department of Neurology, Eskisehir 26080, Turkey; drgulguncu@gmail.com

**Keywords:** paraneoplastic cerebellar degeneration, ataxia, papillary thyroid carcinoma

## Abstract

Paraneoplastic cerebellar degeneration (PCD) is a rare neuroimmunological disease that may accompany tumors. In this article, we present a patient with progressive gait difficulty who was diagnosed with PCD and, in a rare comorbidity, with papillary thyroid carcinoma (PTC) following malignancy screening. A 46-year-old male patient reported having experienced poor balance for 2 years. A neurological examination revealed nystagmus, intention tremor, and ataxia, and anti-thyroid peroxidase and anti-thyroglobulin levels were found to be elevated. A brain MRI showed significant cerebellar atrophy in the superior vermis. Malignancy screening for PCD was performed, and thyroid ultrasonography revealed a nodule in the left lobe, while PET/CT detected elevated focal F-18 fluorodeoxyglucose uptake in the thyroid. Onconeuronal antibodies (anti-Hu, anti-Yo, anti-Ri, anti-amphiphysin, anti-Tr, anti-PPCA-2, anti-Ma, anti-CV-1, and anti-ANNA-3) were negative. Pathologic examination of the thyroid revealed PTC, for which the patient was treated with 0.4 g/kg intravenous immunoglobulin and referred to the medical oncology department. This case demonstrates that clinicians must be alert to the rare comorbidity of PCD and PTC.

## 1. Introduction

Paraneoplastic neurological syndromes (PNSs) are diseases associated with malignancy that involve the nervous system (central, peripheral, and autonomic), which is affected by the immune system through different immunological pathways. PNSs are not triggered by direct tumor invasion, metastasis, or the side effects of treatment. Paraneoplastic cerebellar degeneration (PCD) is a very rare neuroimmunological PNS that may accompany tumors and is often associated with humoral factors that are secreted by tumor cells or with an immune response to tumor cells. It is mostly associated with gynecologic tumors, lung cancer, and Hodgkin’s lymphoma. Acute or subacute onset, moderate or severe truncal ataxia, nystagmus, vertigo, dysarthria, and diplopia may occur [1], and neurological symptoms can be present in patients with PNS before an underlying tumor is detected or may coincide with a cancer diagnosis. The diagnostic criteria for PNS were divided into “definite” and “probable” categories by Graus et al., and the present diagnostic criteria, which may help neurologists to report patients with PNS more uniformly, are based on the type of clinical syndrome (classical or non-classical), the presence or absence of cancer, and onconeural antibodies [2].

In this article, we present a patient with progressive gait and balance difficulties who was diagnosed with PCD and a rare comorbid papillary thyroid carcinoma (PTC) following a malignancy screening.

## 2. Case Description

**Figure 1:**
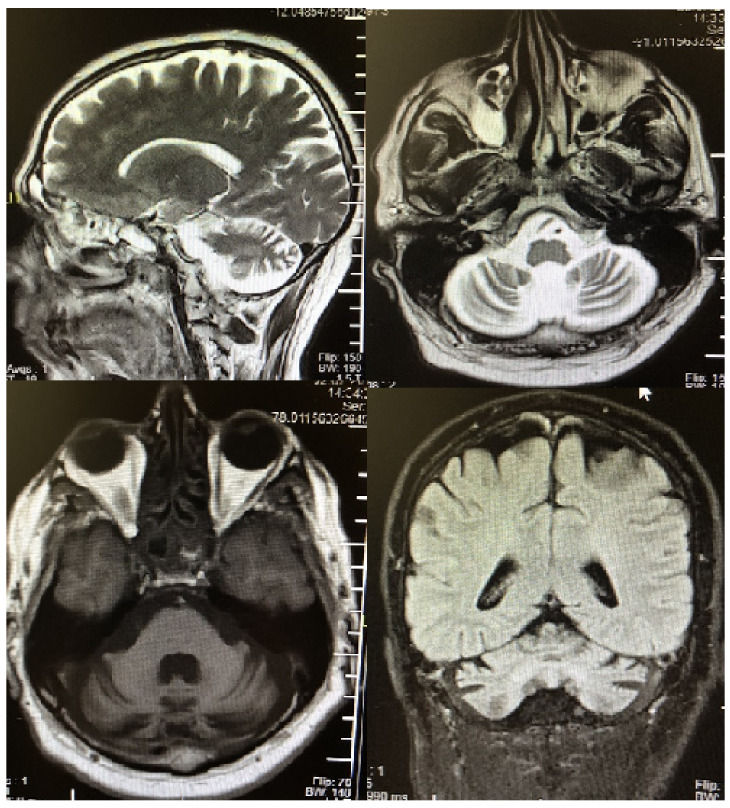
Brain MRI revealed a significant cerebellar atrophy in the superior.

A 46-year-old male patient presented with a gait disorder and reported having experienced poor balance for 2 years. The patient had no known systemic diseases, was a smoker, and his family medical history included no similar abnormalities. Neurological examination revealed horizontal nystagmus, intention tremor, and ataxic gait, and his score on the Scale for the Assessment and Rating of Ataxia (SARA) was 24/40 (gait: 6; stance: 3; sitting: 2; speech disturbance: 4; finger chase: 2; nose-finger test: 2; fast alternating hand movement: 2; heel-shin slide: 3). His routine hemogram and biochemical tests were normal, and, while thyroid function tests were normal, anti-thyroid peroxidase and anti-thyroglobulin levels were elevated. The patient’s cerebral MRI showed a significant bilateral cerebellar atrophy in the superior vermis (Figure 1). Electroneuromyography was normal, and other known causes of the ataxia were excluded (vitamin E, vitamin B12, coenzyme q10, viral infections, genetic tests as an spinocerebellar ataxia, Friedreich’s ataxia and whole exome sequencing).

**Figure 2:**
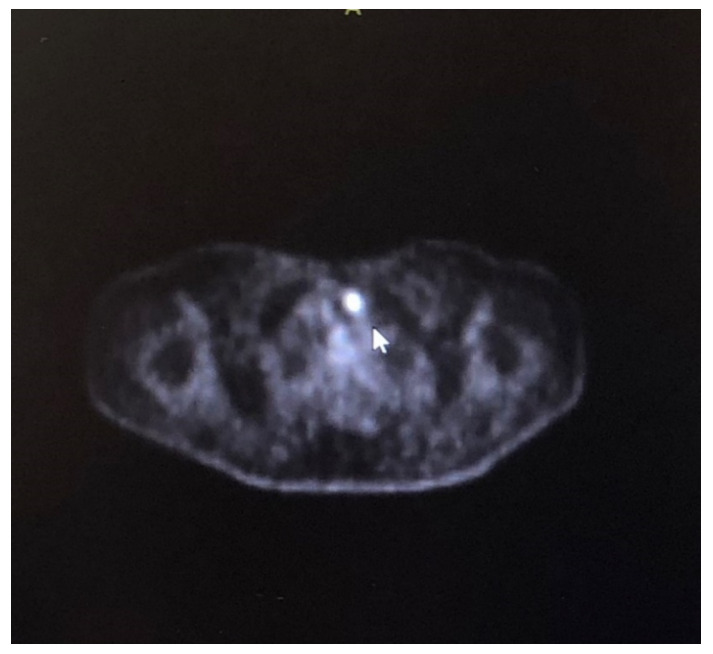
Focal increased F-18 fluorodeoxyglucose uptake was detected in left lobe of thyroid gland on positron emission tomography.

Malignancy screening (thorax, abdominal CT, prostate, and thyroid ultrasonography) for PCD was performed; thyroid ultrasonography revealed a solitary hypoechoic nodule of 11 × 9 mm in the left lobe isthmus junction, and PET/CT detected elevated focal F-18 fluorodeoxyglucose uptake in the left lobe of the thyroid (standardized uptake volume (SUV) max: 7.22; late SUV max: 6.42) (Figure 2). Cerebrospinal fluid (CSF) was examined and found to be normal, and onconeuronal antibodies (anti-Hu, anti-Yo, anti-Ri, anti-amphiphysin, anti-Tr, anti-PPCA-2, anti-Ma, anti-CV-1, and anti-ANNA-3) were negative. A biopsy of the nodule showed that it was consistent with category V (suspected papillary carcinoma) of the Bethesda system [3]. A bilateral total thyroidectomy was therefore performed, and the pathologic examination revealed PTC. The patient was therefore diagnosed with “definite” PNS according to the diagnostic criteria [2] (a classical syndrome, with cancer that develops within 5 years of the diagnosis of the neurological disorder and where the presence of onconeural antibodies is not required).

The patient was then treated with 0.4 g/kg intravenous immunoglobulin for 10 days, following which partial improvement of the ataxia was detected by a SARA score of 20/40 (gait: 4; stance: 2; sitting: 2; speech disturbance: 3; finger chase: 2; nose-finger test: 2; fast alternating hand movement: 2; heel-shin slide: 3). The patient was then referred to the Department of Medical Oncology, and radioactive iodine treatment was started by nuclear medicine during follow-up. When permanent hypothyroidism occurred, thyroid replacement therapy was also started. The patient’s SARA score was still 20/40 after 6 months of follow-up.

## 3. Discussion

In cancer patients, tumor invasion, the effects of metastatic lesions on tissues, infections, and treatment-related side effects are well known. However, PNSs—which are not related to the tumor itself or to its metastasis, but which are dependent on the presence of the tumor—are becoming more frequently recognized. Several hypotheses regarding the pathogenesis and pathophysiology of PNSs have been proposed, but those based on hormones, peptides, and long-/short-acting signaling molecules being produced by tumor cells or through immune reactions with tumor-related antigens are most widely accepted [4]. Increased cerebellar inflammation and cytokine production have been reported in PCD mouse models, and this inflammatory response has been reported to trigger Purkinje cell death even at very early stages (6 days in anti-Yo-associated models) [5,6], with decreases in Purkinje cell diffusion and granule and basket cell loss being detected in the cerebellar cortex.

PCD is a rare PNS; while cerebellar degeneration is acute or subacute at onset due to diffuse Purkinje cell death, it progresses with a rapidly progressing PNS. There are two reported cases with PCD and PTC, which is a rare association in literature. One of the cases, featuring a 57-year-old female patient, was reported by Kroiss et al. [7]. Gratwicke and et al. reported a case with PTC, PCD, and sensory ganglionopathy [8]. In our case, the patient was diagnosed with PCD and PTC at a younger age than the previous cases in the literature.

The symptoms of PCD can be variable. Moderate-to-severe truncal ataxia, nystagmus, vertigo, dysarthria, and diplopia may be observed [9]; in our case, there was a progressive gait disorder. CSF examinations are usually normal at baseline, but inflammatory findings may appear in the analysis—in one study, it was reported that 73% of patients with PCD had inflammatory CSF findings [9]. In our case, CSF findings were within normal limits.

MRI examination of PCD may be normal or reveal severe cerebellar atrophy—one study reported that 75% of patients with PCD had normal MRI findings [9]—and radiographic changes in cerebellar size are usually seen in the late stages of the disease. The patient with PTC, PCD, and sensory ganglionopathy reported in the literature had normal brain MRI findings [8], but we believe that our patient had severe bilateral cerebellar atrophy because he was admitted to hospital for ataxia in the late stage of the disease.

PCD is often associated with gynecologic tumors, breast cancer, lung cancers, and Hodgkin’s lymphoma [10,11,12]. While tumor comorbidity was reported in 42 (84%) patients in a study of 50 patients with PCD [12], comorbidity of PCD with thyroid carcinoma is very rare and has been reported in only two cases in the literature [7,8]. The neurological findings of PCD often appear before the diagnosis of cancer; in a study by Sham et al., 62% of the patients were diagnosed with PCD before a tumor diagnosis [12], and in another study, neurological findings of PCD were observed in 77% of cases before a diagnosis of breast cancer [13]. In the literature, one patient who was diagnosed with renal cell cancer had been diagnosed with PCD 6 years before the first urological findings [14], and, in our case, the patient was diagnosed with PCD after 2 years of reported balance problems, and the diagnosis of PTC was made while the malignancy was being investigated.

Antineuronal antibodies associated with PCD are anti-Yo, anti-Tr, anti-Hu, anti-Ma2, and anti-Ri, and the most common and best described syndrome associated with anti-Yo is PCD. Neurological diseases have been reported to progress rapidly in rare antibody-negative PCD cases [15], and the onconeuronal antibody test was negative in our case. Although these antibodies strengthen the diagnosis of autoimmune paraneoplastic syndrome, a failure to detect them does not exclude the diagnosis, as in our patient.

PCD usually has a poor prognosis, but rapid and effective treatment can prevent the progression of symptoms. Tumor therapy has been reported to be more beneficial than immunotherapy in stopping neurological progression [12]; in a follow-up of 12 patients with PCD and breast cancer, the mean survival was 100 months, and five (42%) of the patients died [16]. In our case, the patient diagnosed with PTC received surgical therapy and medical oncologic control. The patient is still being treated with thyroid hormone replacement following iodine therapy.

As in our case, patients with no known tumor diagnosis may present with the clinical symptoms and signs of PCD. The presence of a tumor can be shown through etiological investigation of PCD. Thus, the earlier a tumor is detected, the higher the likelihood of tumor and PCD treatment. Clinicians should be alert to patients with progressive gait disorder as a PCD symptom, which can be comorbid with PTC.
